# How adults with cerebral palsy successfully confront and cope with ableism: a peer-led research project

**DOI:** 10.1080/17482631.2026.2616117

**Published:** 2026-01-15

**Authors:** Cadeyrn J. Gaskin, Andrew D. Brown, Sue Harris, Alex Birnie, Carmen Vargas, Finn O’Keefe, Angela Dew, Debbie Dorfan, Freya E. Munzel, Claudia Strugnell, Maddie Fogarty, Adam Goodridge, Joy Martin Mitchell, Shelley Spencer

**Affiliations:** a Institute for Health Transformation, Deakin University, Geelong, Australia; b School of Health and Social Development, Deakin University, Geelong, Australia; c Cerebral Palsy Support Network, Melbourne, Australia; d Institute for Physical Activity and Nutrition, Deakin University, Geelong, Australia; e School of Exercise and Nutrition Sciences, Deakin University, Geelong, Australia

**Keywords:** Ableism, cerebral palsy, confronting, coping, defence mechanisms, disability, discrimination, microaggressions, prejudice, stereotype

## Abstract

**Purpose:**

This study focused on how adults with cerebral palsy successfully confronted ableism during encounters with others and successfully coped with ableism in general.

**Methods:**

Adults with cerebral palsy led this critical participatory action research project, in which ten adults with cerebral palsy shared their experiences (via an online survey or interview) of successfully confronting ableism (situations, actions taken, and outcomes) and coping with ableism.

**Results:**

Participants had difficulty recalling successful confrontations due to failing to recognise ableism, ignoring it, or being unsure whether confrontations were successful. Of the 23 situations described, common forms of ableism were denial of privacy, perceived helplessness, and spread effect. Actions taken in successful confrontations were educating perpetrators, being independent, self-advocating or requesting advocacy, attempting to make perpetrators feel uncomfortable, and disengaging with perpetrators (and encouraging others to do similar). Outcomes were changed perpetrator behaviour, apparent changed perpetrator perceptions, actions to prevent recurrence of ableism, disengagement, changed thinking, and feeling successful. Adults coped with ableism through changing their own thinking about disability and ableism, engaging in everyday activities, seeking social support, and making efforts to change society.

**Conclusions:**

Harnessing this knowledge may assist people with cerebral palsy to challenge the social oppression they face.

## Introduction

Prejudice and discrimination against people with disability based on the assumed superiority of non-disabled people and their ways of doing things—known as ableism—is deeply embedded within society (Charlesworth & Banaji, 2019; VanPuymbrouck et al., 2020; Wilson & Scior, 2014). Projections from the US suggest that it may take over 150 years for implicit (unconscious) bias against people with disability to neutralise (Charlesworth & Banaji, 2019). People with cerebral palsy are particularly affected. Evidence shows that attitudes towards people with cerebral palsy are more strongly negative than for people with many other types of impairments, such as people with amputations, sensory impairments, and paraplegia (Mastro et al., 1996; Olkin & Howson, 1994; Westbrook et al., 1993). One of the ways in which ableism manifests is in everyday encounters with others.

Ableism in encounters with others ranges from seemingly benevolent (e.g., pity, paternalistic protection, and unwarranted praise) to hostile (e.g., avoidance, gestures of disgust, hate speech, aggression, and violence) (Burch, 2018; Hughes et al., 2012; Jones et al., 2012; Kouznetsova et al., 2012; Nario-Redmond et al., 2019). Often ableism presents in ambivalent forms, such as when pity and paternalism turn to hostility when offers of assistance are declined (Nario-Redmond et al., 2019). Commonly reported forms of everyday ableism include paternalism (e.g., unwanted help, infantilisation, general pity, invalidation of impairments, and overprotective families), admiration (e.g., being referred to as inspirational for undertaking everyday tasks), hostility or anger (e.g., verbal abuse and ridicule, physical or sexual assaults, and general harassment), envy or jealousy (e.g., due to disability-related accommodations, such as accessible parking), dehumanisation or objectification (e.g., depersonalisation, physical invasions of privacy, and abandonment or neglect), and fear (e.g., expressions of a preference for death over disability and of a concern of catching disability or passing it on to offspring) (Nario-Redmond et al., 2019). The visibility of impairments seems to matter. People with visible impairments (often the case with cerebral palsy) report certain types of ableism (e.g., unwanted help, overprotective families, infantilisation, being referred to as inspirational, and physical invasion) more frequently than those with less apparent impairments, whereas those with less apparent impairments have a greater tendency to report other types of ableism (e.g., doctors, family, and friends questioning the legitimacy of their conditions; being perceived as “exploiting the system”) (Nario-Redmond et al., 2019).

Although ableism is sometimes overt, it can also take the form of microaggressions (Keller & Galgay, 2010)—brief, often automatic, seemingly innocuous, everyday exchanges that denigrate people due to their disability (Keller & Galgay, 2010; Pierce et al., 1977; Steele, 2017). Often people act with good intent, but their actions reveal prejudices against people with disability. Examples of microaggressions towards people with disability are plentiful (see Table I) (Keller & Galgay, 2010; Olkin et al., 2019). Ableist microaggressions are harmful (i.e., annoying, disrespectful, hurtful, or insulting), particularly those perpetrated by family and friends (Conover et al., 2021; Olkin et al., 2019). More frequent lifetime experiences of ableist microaggressions are associated with greater depressive symptoms (Conover & Israel, 2019; Conover et al., 2021). The effects of ableism on health and well-being may be different to other forms of discrimination (e.g., sexism and racism), possibly due to environmental barriers, including prejudice and discrimination, contributing to social isolation and limited social support (Branco et al., 2019).

**Table I.** Examples of ableist microaggressions*.*

Type	Meaning
Denial of disability	Denying or minimising disability-related experiences.
Denial of personal identity	Overemphasising disability and ignoring other salient aspects of personal identity.
Denial of privacy	Demanding personal information from a person with disability, or unnecessarily touching their body or equipment.
Helplessness	Offering or providing help to a person with disability without asking when it is not requested nor needed.
Infantilisation	Treating a person with disability like a child or young person.
Patronisation	Being condescending or praising a person with disability for doing everyday tasks.
Second-class citizenship	Denying the right to equality because respecting the right is considered bothersome, unjustified, or unreasonable.
Secondary benefits	Having expectations of praise or feeling good for doing something for a person with disability.
Spread effect	Making assumptions that functional limitations in one area translate to limitations in other areas.

Note: Examples drawn from Keller and Galgay (2010) and Olkin et al. (2019).

Confronting prejudice is an active coping strategy (Chaney et al., 2015) that may have the potential to reduce prejudice (Chaney & Sanchez, 2018; Czopp et al., 2006) and to improve the mental health among those who experience prejudice (Foster, 2013; Foster, 2015; Sanchez et al., 2016). Confrontations can take many forms, including speaking to the perpetrator directly, reporting the incident to someone in authority (e.g., human resources personnel), or responding non-verbally (e.g., eye rolls, offensive hand gestures) (Chaney & Sanchez, 2022; Good et al., 2022). An example of a conceptual model in this space is the Confronting Prejudiced Responses Model (Ashburn-Nardo et al., 2008). According to this model, there are at least five hurdles that must be met for prejudice to be confronted: (1) an incident needs to be interpreted as discrimination, (2) the incident needs to be considered an emergency (i.e., harmful enough to warrant intervention), (3) the target of prejudice (or observers) needs to take responsibility for saying or doing something, (4) the target (or observers) needs to know how to respond, and (5) the target (or observers) needs to decide to take action. The effectiveness of confrontations has been associated with a range of factors, such as who does the confronting (e.g., non-target confronters are perceived more favourably than target confronters (Gulker et al., 2013; Rasinski & Czopp, 2010), who can be seen as complaining (Gulker et al., 2013) and overreacting (Czopp & Monteith, 2003; Kaiser & Miller, 2003), where the confrontation occurs (e.g., in organisational settings, public confrontations may be viewed more favourably than those occurring in private (Gervais & Hillard, 2014), and what is said (e.g., hostile accusations may be as effective as appeals for fairness and equality, but generate stronger feelings of anger towards confronters (Czopp et al., 2006). Evidence also suggests that the type of prejudice matters, with racism concerning people more (Gulker et al., 2013) and evoking more guilt and apologetic responses (Czopp & Monteith, 2003) than sexism. From the limited amount of research on confronting ableism, people who use wheelchairs and, especially, those who are blind were perceived as less warm and ruder after they confronted help that was clearly patronising (Wang et al., 2019). Whereas both people who were blind and those who were sighted considered hostile treatment inappropriate (e.g., refusing to answer a question about directions), people who were sighted perceived benevolent treatment (e.g., taking a person by the arm, without consent, and insisting on taking them to their destination) as more appropriate than those who were blind (Wang et al., 2015). With much of the research on confronting prejudice focused on racism towards Black people and sexism towards women (Monteith et al., 2019), most studies being conducted in artificial settings (e.g., survey responses to vignettes and laboratory experiments), and evidence that people’s reactions to prejudice depends on who is targeted (Czopp & Monteith, 2003; Gulker et al., 2013), there is a need to understand how ableism can be confronted in the real world.

Contending with ableism not only requires ways of confronting prejudice when it occurs but also strategies for coping with the enduring effects of living in ableist environments. As proposed foundations for work in this area, general models of stress and coping (e.g., transactional model of stress and coping (Lazarus & Folkman, 1984) and a general conceptual framework of the coping process (Moos & Schaefer, 1993) have been extended to stigma and discrimination (Berjot & Gillet, 2011; Miller & Kaiser, 2001). In these frameworks, personal factors (e.g., hypervigilance to rejection and coping styles) and situational characteristics (e.g., social context and social support) are antecedents to stress and coping. One of these models has also drawn attention to the characteristics of stigma—particularly its visibility and controllability—as antecedents (Berjot & Gillet, 2011). Whether or not an event is perceived as stressful depends on a cognitive appraisal of the situation (Berjot & Gillet, 2011; Miller & Kaiser, 2001). An event will be stressful if it is perceived as a threat (primary appraisal) that could exceed available resources for coping (secondary appraisal). An inability to cope with stigma-related events is detrimental to well-being. Alongside the increasing evidence of the adverse consequences of discrimination, such as racism (Williams et al., 2019), on mental and physical health, there is emerging evidence of the harmful effects of ableism on health and well-being (Branco et al., 2019).

Possible methods of coping with stigma and discrimination can be classified in different ways (Berjot & Gillet, 2011; Miller & Kaiser, 2001; Nario-Redmond, 2020). One proposed way of understanding how people cope with ableism centres on social mobility strategies (e.g., endeavouring to remove or conceal impairments, or trying harder to meet the norms of broader society), social creativity strategies (e.g., revaluing impairments as assets), and social change strategies (e.g., forming advocacy groups or engaging in collective action) (Nario-Redmond, 2020). The importance of social change strategies with this framework is that they signify a shift from individual-level understandings of stress and coping towards recognising systems and structures that produce and sustain discrimination, which can be interrogated using critical theoretical perspectives (Varcoe et al., 2019). People with disability tend to differ in their propensities to use each strategy, with, for example, social change strategies being most likely to be used by (a) those with more severe, enduring, or visible impairments; (b) those who identify highly with disability; and (c) those who view the stigma they experience as illegitimate, unjustified, and modifiable (Nario-Redmond, 2020).

Although both individualistic (social mobility) and collectivistic (social creativity and social change) coping strategies are incorporated within the framework (Nario-Redmond, 2020), the potential role of adaptive defence mechanisms in coping with ableism is overlooked. Alongside social support and conscious cognitive strategies, defence mechanisms is one of three broad classes of coping mechanisms within the psychological literature (Vaillant, 2000). Based on their usual level of adaptiveness (extent to which they improve adaptation to stressors), defence mechanisms have been organised hierarchically, with *high adaptive defences* (affiliation, altruism, anticipation, humour, self-assertion, self-observation, sublimination, and suppression) the most adaptive (Di Giuseppe & Perry, 2021; Perry & Bond, 2012; Perry, 2014).

Given the strength of negative attitudes towards people with cerebral palsy that exist within society (Al-Dababneh & Al-Zboon, 2018; Mastro et al., 1996; Smith et al., 2025), there would seem to be merit in directing research attention towards supporting them to confront and cope with ableism. In this regard, people with cerebral palsy themselves can be a fruitful source of solutions to the challenges they face. Grounded in the experiences of adults with cerebral palsy, previous research has identified common themes in how they have achieved successful outcomes across many areas of life, such as education, employment, independent living, and close relationships (Gaskin et al., 2021). Ableism represents another challenge. Understanding how some adults with cerebral palsy confront and cope with ableism could inform strategies to support others to do the same. Building upon the strength-based, solution-focused approach used in this prior research (Gaskin et al., 2021), we sought to understand how adults with cerebral palsy manage and cope with ableist encounters. Specifically, we investigated how adults with cerebral palsy have successfully confronted ableism during encounters with others and how they have successfully coped with ableism in general.

## Methods

### Approach

Engaging with a critical qualitative research paradigm, the ultimate goal of this work was for people with cerebral palsy to challenge the social oppression they face through generating and disseminating knowledge on successfully confronting and coping with ableism. Critical qualitative research has firm roots in a human rights agenda, using transformative inquiries to challenge human oppression, inequality, injustice, and poverty in the pursuit of social justice (Denzin, 2016). Specifically, we undertook critical participatory action research (Kemmis et al., 2014), in which people with cerebral palsy led and were co-researchers in the project. Our team formed voluntarily and comprised five academic co-researchers (one of whom has cerebral palsy) and 11 peer co-researchers (adults with cerebral palsy affiliated with Cerebral Palsy Support Network [CPSN]) who committed to pursuing peer-led research on a matter of importance to adults with cerebral palsy. We use the terms *academic co-researchers* and *peer co-researchers* to reflect the distinct but equally valued forms of expertise within our team—formal academic training and lived experience of cerebral palsy. The academic co-researchers had backgrounds in various disciplines (psychology, sociology, health promotion, social work, and education) and qualitative research expertise. The peer co-researchers had various experiences with research, including as participants, researchers (consumer and co-researchers, an associate investigator, and a co-ordinator), and advisors (a technical advisor, a consultant, and advisory and steering committee members).

In our team, power was shared and all co-researchers contributed to shaping the research aims, design, and outcomes. Discussions were held online in the early evening to accommodate the schedules, accessibility needs, locations, and preferences of the co-researchers. With leadership from CJG (person with cerebral palsy, Deakin University senior research fellow, and former CPSN board director), academic co-researchers facilitated group discussions during which the peer co-researchers determined the topic of the research and decided upon the research questions. Peer co-researchers provided guidance on how the project should be conducted, including suggesting that multiple data collection options should be made available to potential participants. Several peer co-researchers (FO’K, AB, AG, DD, and FEM) assisted in developing and reviewing the recruitment material, including appearing in the recruitment video. Their contributions strengthened the explanation of ableism in the material, with the addition of multiple illustrative examples. CJG collected the data. AB (a peer co-researcher) and academic co-researchers (CJG, ADB, and CV) transcribed the interviews. CJG led the initial steps of the data analysis, with AB assisting with the coding of some of the interviews. As a group, we discussed and finalised the data analysis, with peer co-researchers making changes to the construction and naming of themes. Through these discussions, the peer co-researchers also enhanced the interpretation of the findings, and provided guidance and feedback on the reporting and dissemination of the research.

We acknowledge that our prior experiences, assumptions, and beliefs have shaped the research, and have each provided a reflexivity statement in Supplementary Table I. Briefly, all of the adults with cerebral palsy in our team had experienced ableism. Several of our team had experienced other forms of prejudice and discrimination, mainly due to sexual or gender diversity. At the beginning of the project, the understanding of ableism within our team varied substantially.

### Design

People with cerebral palsy differ in their communication strengths. Whereas some find it easiest to communicate verbally, others use assistive technology or prefer written communication. To accommodate these differences, we provided several ways of participating: online survey, individual interview (online or in person), online focus group, and self-recorded video or audio submission.

Whichever option they chose, participants were asked to respond to the same questions. An overview of the survey and interview questions is provided in Table II. Briefly, participants were asked for examples of when they had successfully confronted ableism, describing the situations, what they did, and the successful outcomes. Participants were encouraged to provide as many examples as they could. They were then asked, in general, how they successfully coped with ableism (i.e., in the moments, days, weeks, or years after the encounters). We also asked participants to report their gender and age range.

**Table II.** Overview of survey and interview questions*.*

Topic	Questions
Confronting ableism	**How have you successfully confronted ableism in encounters with others?** We would like examples of when you have successfully confronted ableism. For each example, please describe: the situation, what you did, and the successful outcome.
Coping with ableism	**In general, how do you successfully cope with ableism?** In answering this question, it can be useful to reflect on what you do (either individually, with others, or both) and how you think.
Demographics	**Which gender do you identify as?** (female, male, another gender you specify) **Which age range applies to you?** (18−24, 25−34, 35−44, 45−54, 55−64, 65+ years)

Deakin University Human Research Ethics Committee granted approval for this project to be undertaken (project number: 2023-126). All participants provided written or verbal (audio-recorded) informed consent prior to their participation.

### Participants

Adults (≥18 years) with cerebral palsy who self-identified as being able to describe experiences of successfully managing ableist encounters or coping with ableism were eligible to participate in the study. What *success* meant was for each participant to define. In previous research, success has been variously defined in terms of perpetrator behaviour (e.g., reduced use of stereotypes (Chaney & Sanchez, 2018; Chaney et al., 2021; Czopp et al., 2006) and the match between desired and actual perpetrator behaviour (Good et al., 2022). Confronting prejudice has also been associated with enhanced psychological well-being among confronters, including greater competence (Gervais et al., 2010), satisfaction with their responses (Hyers, 2007), self-esteem (Gervais et al., 2010), autonomy (Sanchez et al., 2016), and empowerment (Gervais et al., 2010; Hyers, 2007), as well as less need for social support (Hyers, 2007). Conversely, not confronting prejudice has been associated with negative reactions, such as feelings of guilt, regret, and disappointment (Shelton et al., 2006).

The approach taken in this research (i.e., critical participatory action research, multiple ways of participating, and an interpretative form of data analysis) made it challenging to estimate the required sample size. Whereas saturation (Hennink & Kaiser, 2022; Malterud et al., 2015) may be a useful guide for (post) positivist projects, it is not considered appropriate for reflexive thematic analysis (Braun & Clarke, 2021). In projects using reflexive thematic analysis, sample size estimations are pragmatic decisions based on considerations such as the time and resources available to the project, the breadth and focus of the research question, the methods of data collection, the identity-based diversity within the population, and the depth of data likely to be collected from individual participants (Braun & Clarke, 2021). We regarded the resources available to this project to be limited, the research questions to be quite narrow, the data collection methods available were broad, the population was narrow in terms of diagnosis but diverse in terms of motor and cognitive function, and the amount of information collected from each participant could be limited. Based on this assessment, we anticipated recruiting 15 to 25 people.

Participants were recruited through organisations that provided support and information to people with cerebral palsy. These organisations distributed advertising in written and video forms. Potential participants were invited to nominate which form of participation best suited them (online survey, individual interview, focus group, or video or audio submission).

Ten adults with cerebral palsy participated in the study. Two were aged between 25 and 34, six between 35 and 44, and two between 55 and 64. There were seven females, two males, and one trans non-binary person. Five completed the online survey and five were interviewed online. Interview durations ranged from 49 to 80 minutes (mean = 60 minutes).

### Analysis

Our analytical approach focused on understanding how adults with cerebral palsy successfully confronted and coped with ableism, with separate analysis and reporting of findings for each. For the analysis of confronting ableism, we separately examined the situations in which ableism was confronted, the actions participants took, the outcomes that followed, and potential links between actions and outcomes. The analysis of situations in which ableism was confronted involved deductive content analysis, in which we classified the situations using the names and definitions of different types of ableism identified in previous research (Keller & Galgay, 2010; Olkin et al., 2019). This classification ensured our nomenclature aligned with the literature. With critical qualitative research as a guide, we then used reflexive thematic analysis (Braun & Clarke, 2006, Braun & Clarke, 2019; Braun et al., 2019) to examine the remaining data on confronting (including actions and outcomes) and coping with ableism. This critical approach was evident in the meaningful involvement of team members with cerebral palsy in interpreting the data, our interpretation of participants’ experiences as instances of ableism, and our acknowledgement that our identities and experiences shaped the construction of themes. Reflexive thematic analysis was well-suited to this research, as it emphasises the active roles of researchers in the production of knowledge, and recognises that researcher subjectivity is a valuable analytic resource (Braun et al., 2019).

Drawing on Braun and Clarke (Braun & Clarke, 2006; Braun et al., 2019), the analysis had six steps: (1) data familiarisation, (2) code generation, (3) theme construction, (4) theme review, (5) theme definition and naming, and (6) report production. CJG undertook the first three steps, with assistance from AB with code generation for two of the interviews. ADB and CV reviewed this initial analysis. The revised analysis was then circulated to the whole team and discussed at a subsequent meeting. This discussion resulted in revisions to the groupings of codes within themes, the merging of themes, and the naming and renaming of themes. CJG used the revised themes to assess whether there were potential links between the actions taken and the outcomes that followed. He then produced a report of the findings from this study. Another whole team meeting was then held to confirm the themes and to discuss the findings and implications. Feedback was integrated into the report, which was circulated for final review and approval.

When presenting quotes, we used square brackets to show where we have replaced participants’ words for clarity. Given the small number of participants and our concern for maintaining anonymity, we chose not to include identifiers with quotes.

## Results

Based on the aims of the study, the findings are presented in two main sections, one on how adults with cerebral palsy successfully confronted ableism during encounters with others, and the other on how they coped with ableism after the encounters.

### Successfully confronting ableism during encounters with others

In responding to the questions on successfully confronting ableism, participants provided contextual information about confronting ableism. The themes we developed from these data are provided first. We then report the findings of the separate analyses on the situations where confrontations occurred, actions taken, outcomes, and links between actions and outcomes.

#### Contextual information

Encountering ableism was common in the lives of participants. The effects of ableism seemed to compound over time, as a participant described.

*I wish I could hit the pause button on ableism for a while and have a break because I feel like it's compounding. And you have the big moments [that] you feel impacted in your life. But you have all the micro, subtle forms that just continue as well. So, I don’t feel like I get to kind of look back as though [ableism] only happened back then.*

Participants generally indicated that they had difficulties providing examples of times they had successfully confronted ableism. These difficulties were attributed to (a) failing to recognise ableism, (b) ignoring ableism, or (c) being unsure as to whether confrontations were successful. A reason given for not recognising ableism was the denial of ones’ own cerebral palsy (i.e., the experience cannot be perceived as ableism if one does not perceive oneself as having a disability). Participants stated that they would typically ignore ableism and block ableist encounters from their memories. They also indicated that determining the outcomes of confronting ableism was difficult and that confronting ableism is generally unsuccessful.

*It's hard to think of, like, successful [examples of confronting ableism] because I'd say the majority aren't successful.*

Although they were not directly asked, some participants suggested that there were facilitators and challenges to confronting ableism. The two facilitators mentioned were (a) having confidence and (b) having confronted one’s own internalised ableism.

*You can’t confront … the ableism that you face if you can’t confront your disability.*

The most mentioned challenge to confronting ableism was that perpetrators often have good intentions (e.g., referring to people with disability as courageous). Other challenges external to people with cerebral palsy included that strong ableism is difficult to change, perpetrators do not generally understand the concept of ableism, perpetrators are not used to being confronted, and perpetrators make incorrect assumptions about the experiences of disability. Internal challenges to confronting ableism included having self-doubt, failing to recognise ableism, feeling pressure to find ideal responses, and finding it exhausting to have to explain oneself. A question was raised as to whether people with cerebral palsy are responsible for educating others.

*Interviewer: What’s your view on [whether people with cerebral palsy are responsible for educating others]?*

*Participant: Ideally, yes, but a better man than me can do it. I sometimes do it.*

One participant was optimistic about the future, suggesting that the need to confront ableism may reduce over time due to increasing inclusion and acceptance of people with disability in society.

#### Situations where the confrontations occurred

Between them, the participants provided 23 examples of times when they successfully confronted ableism (see Supplementary Table 2). The situations participants described represented eight forms of ableism: perceived helplessness, spread effect, denial of privacy, denial of personal identity, denial of disability, second-class citizenship, patronisation, and secondary benefits. Denial of privacy and spread effect were the most common forms of ableism, accounting for 9 of the 23 examples.

#### Actions taken to confront ableism

There were five main actions that participants took when they successfully confronted ableism: educating perpetrators, being independent, self-advocating or requesting advocacy, attempting to make perpetrators feel uncomfortable, and disengaging with perpetrators (and encouraging others to do similar). Educating perpetrators involved providing explanations and correcting misconceptions. Participants explained about their cerebral palsy and the relationships between people (e.g., the person with cerebral palsy, not the support worker, was the child’s mother). They also corrected misconceptions that their abilities were less than others assumed and that their achievements were exceptional.

*I often respond to such situations by explaining that my achievements are the result of having similar expectations placed on me as my siblings and having access to the necessary support, resources, and structures to meet those expectations. Therefore, my actions aren't exceptional but rather the result of a (mostly) level playing field and the determination to pursue my goals, just like anyone else.*

Being independent included declining assistance from others (e.g., a person with cerebral palsy telling a teacher that she did not need assistance and wanted to perform the task independently) and engaging in tasks independently.

*When [the nurses] watched me change nappies and things, I used my one hand. I used my teeth to kind of hold different things, and it looks a bit messy, but it gets the job done.*

Self-advocating or requesting advocacy involved confronting ableism through speaking up for themselves and gaining advocacy support from others (e.g., parents).

*And, yeah, so I emailed them saying, well [the inaccessible stage is] still not really good enough and we kind of went back and forth.*

Attempting to make perpetrators feel uncomfortable included refusing to answer questions, giving flippant responses, and asking equally personal questions. Responses ranged from politely refusing to answer questions to giving blunt responses and walking away to delivering humorous, sexualised verbal responses. Participants who were asked personal questions (e.g., how do you go to the toilet?) asked the same questions that they were asked or retorted with equally personal questions.

*And you know, they do that thing where they come and put their hand on your thigh. And kind of lean in a bit. And then be like, “What's wrong with you, love?” And I [said], “I got whiplash from a vibrator, mate.” And they would just, like, the fact that someone with disability could have a wank [and] could say something so out there [it] would just freak them out so much they would leave me the fuck alone.*

Disengaging with perpetrators involved ignoring people’s ableism, avoiding these people, and encouraging others to do similar. Examples included a person ignoring and avoiding a parent who was ableist and another who encouraged a former lecturer not to send students to an employer who was ableist.

*I was just really over it. Whenever I, kind of, spoke to [my father] I just felt like, you know, particularly around my achievements and around what I was doing. Like he would, yeah, make those comments about it being because of my disability and whatever, so I just started to, kind of, really ignore it and cut it out from there.*

#### Outcomes of ableist encounters

There were six main outcomes of the ableist encounters: changed perpetrator behaviour, apparent changed perpetrator perceptions, actions to prevent recurrence of ableism, disengagement, changed thinking, and feeling successful. Changed perpetrator behaviour refers to changes, at the time, in behaviour or commitments to change in future. Examples included perpetrators stopping shouting (assuming the people with cerebral palsy had a hearing impairment), refraining from asking further personal questions, and moving to enable a person with cerebral palsy to exit a carpark. The following quote refers to the outcome of a situation where a customer tried to help a person with cerebral palsy to enter her personal identification number when making a purchase, and the person with cerebral palsy informed the customer that she was capable of entering the number and requested space to do so.

*The other customers are left with no choice but to back away.*

Apparent changed perpetrator perceptions refers to shifts in perpetrators’ understanding of cerebral palsy and what people with cerebral palsy are capable of doing. Many examples were of perpetrators with seemingly low expectations of people with cerebral palsy who were surprised with what they were able to achieve.

*And you know, you kind of get this look from [the nurses] like, oh my god, what's going on here? Oh, all right, yep, she's changing the nappy, yep, we'll leave her to that. So yeah, I think in the situation with the second and third [babies] it was through … them experiencing the way that I did things. For them to see that maybe their first thoughts around me not being able to do things [were] not correct. I can do [the nappies], I just do them in a different way.*

Actions to prevent recurrence of ableism involved perpetrators being confronted sometime after the situation where the ableism occurred and remedial actions being taken. Examples included parents confronting a school principal, and students receiving education about cerebral palsy and being asked to apologise for their ableism.

*I remember the girls (five of them) were all called into a meeting with our class teacher, and they were forced to write notes of apology to me.*

Disengagement involved people with cerebral palsy or perpetrators (or both) physically removing themselves from situations where ableism occurred or people with cerebral palsy suggesting to others that they should consider disengaging with the perpetrator.

*And so sometimes success looks like allowing myself to turn around and wheel away.*

Changed thinking refers to gaining insights into ableism and how to handle it and overcoming another person’s ableism. Insights gained included realising that sometimes another person’s ableism cannot be changed and that people are not inherently bad but need educating.

*Maybe it's come with time. I don't think people are inherently bad or mean. They just don't know and it's scary when you don't know, when you don't understand something. So, where I'm able to provide information that might make them feel a bit more secure in themselves, in their knowledge, then that's what I'll try to do.*

Feeling successful manifested in several forms including feeling good about explaining cerebral palsy, not answering perpetrators’ questions, remaining calm and in control when responding to perpetrators, and feeling that it was a successful outcome.

*For me, I suppose, I don’t think it'll change people's behaviour. I think. Or attitude. I think it's in that situation. It’s about, for me, me wanting to maintain control of that situation. So, I think more often than not, I react negatively. If I can react and stay calm and call it out, then I feel at peace with myself. I think it’s about feeling at peace with myself.*

#### Links between actions and outcomes

In the examples of confronting ableism that participants described, certain actions appeared to be linked with specific outcomes. Educating perpetrators and being independent seemed to be linked to changed perpetrator behaviour and apparent changed perpetrator perceptions. Attempting to make perpetrators feel uncomfortable seemed to be connected to disengagement and feeling successful. Other actions (self-advocating or requesting advocacy, and disengaging with perpetrators) seemed to be linked to multiple outcomes.

### Coping with ableism

There were four main ways in which people with cerebral palsy successfully coped with ableism: changing their own thinking about disability and ableism, engaging in everyday activities, seeking social support, and making efforts to change society. Changing their own thinking manifested in different ways. The presence of high adaptive defensive mechanisms seemed evident, with participants speaking about blocking ableist encounters from their memories, using humour (e.g., seeing the silliness in some forms of ableism), and turning to others for support. Participants spoke of the benefits of learning about disability rights, the social model of disability, the ways in which ableism manifests, and the ableism experiences of other people with disability. Cognitive reframing was sometimes used, such as perceiving society as the problem (not the person with disability), viewing ableism as a teachable moment, seeing confronting ableism as an opportunity to reduce the chances of others experiencing ableism, and looking upon ableist experiences as sources of material to teach others about confronting ableism. Responding positively when experiencing ableism was suggested as a way of enhancing self-esteem. Changing their own thinking also involved identifying as a person with cerebral palsy and, more broadly, a person with disability.

*The shift for me in terms of how I felt about myself and how I felt about the ableism that I was encountering as a young person, the shift from feeling like I was wrong and I was flawed and there was something wrong with me was when I got that political analysis of the social model and disability as a human rights issue and started to think, “No hang on, the problem isn't me. The problem is ableism and structural issues in society that exclude disabled people.” And that was really pivotal, pivotal in changing my feelings about myself and my feelings about the world.*

Engaging in everyday activities was a way of successfully coping with ableism through living life as if they did not have cerebral palsy (while being mindful to request support when needed) and demonstrating that they are more than their disability. Participants seemed keen to project that they have a range of talents and interests, rather than being seen as one-dimensional (a person with cerebral palsy).

*I've spent the past week or so just training in this video game so that I can get better at it, so that I can kind of feel like, “Oh, I did this” and it's not necessarily to prove people wrong or anything like that, but just to kind of be like, I'm more than my disability.*

Seeking social support was a way of coping with ableism. Participants sought social support from others who have experienced oppression, including people with cerebral palsy, people with disability, and others (e.g., some queer and trans folk). They spoke about how being around others with cerebral palsy served to normalise and validate their experiences and gave them a deep connection with people with cerebral palsy. Participants gained support from their families and friends, either in person or via social media. They also sought counselling from professionals who were disability aware or open to learning about disability. Participants mentioned that they needed to be proactive when choosing a counsellor, because they found that some counsellors were ableist.

*So having a sense of community around me and sometimes that's even about like when something happens and I'm just like oh for fucks sake, being able to write a Facebook status update about it and get some support, like immediately, in those moments or writing a humorous thing about it. And knowing that, you know, I've got that virtual community who get it. … So sometimes it's not just about having those physical people in your life. When you're dealing with it, with those kinds of shitty responses or perceptions, also having that virtual community as well has been really important.*

Making efforts to change society as a way of successfully coping with ableism involved educating others about disability, teaching people with cerebral palsy how to confront ableism, and engaging in disability advocacy.

*I used to avoid being seen as a person with a disability. Now I have learnt to embrace it and believe it is essential to change society and often that happens by educating one person at a time.*

## Discussion

With ableism being a challenging and often harmful constant in the lives of many people with cerebral palsy, the evidence contains a powerful message that, in some instances, such behaviours may be able to be successfully confronted and there may be ways of successfully coping with the enduring effects of these encounters. From examples of a broad range of situations, we identified five actions that participants described as successful in confronting ableism: educating perpetrators, being independent, self-advocating or requesting advocacy, attempting to make perpetrators feel uncomfortable, and disengaging with perpetrators. We also identified four ways that participants were successful in coping with ableism over time: changing their own thinking about disability and ableism, engaging in everyday activities, seeking social support, and making efforts to change society.

With previous research showing that confronting may have the potential to reduce prejudice (Chaney & Sanchez, 2018; Czopp et al., 2006), our findings suggest that certain actions may be more likely to achieve this outcome. Educating perpetrators and being independent seemed to be linked to potential reductions in prejudice (manifesting, in our study, as changed perpetrator behaviour and apparent changed perpetrator perceptions). Gaining a greater understanding of cerebral palsy and observing people with cerebral palsy functioning beyond their expectations may be sufficient for some perpetrators to reflect on their own assumptions and adjust their thoughts and behaviours (at the time, at least).

Confronting may also have the potential to improve mental health (Foster, 2013; Foster, 2015; Sanchez et al., 2016). Although not explicitly explored with participants in this study, all five actions may have positive mental health benefits and warrant further investigation. Taking action may assist in developing a sense of competence in managing ableist encounters. Competence is regarded as a core component of positive self-esteem, which, in turn, is associated with mental well-being, success, satisfaction, adjustment, and happiness (Mann et al., 2004). That some participants felt satisfied with the actions they took may tentatively suggest an increase in self-esteem and a strengthened sense of competence.

The diversity of actions reflect that different responses may be more successful in some situations and less successful in others. Participants suggested that the perceived gaps between their understandings of cerebral palsy and those of perpetrators was one of the challenges to successful confrontations. Educating perpetrators might be less successful when there may be a sizable gap between perpetrators’ beliefs about disability and the experiences and understandings of people with cerebral palsy. In such situations, responses that potentially preserve or enhance the mental health of people with cerebral palsy (e.g., attempting to make perpetrators feel uncomfortable or disengaging with them) may be the more successful option.

Rather than striving to conform to dominant norms (social mobility), participants engaged in coping strategies that affirmed disability as valuable (social creativity), pursued systemic transformation (social change), and demonstrated high adaptive defences. Social mobility strategies (attempting to *escape* disability through medical intervention, denying or concealing disability, or embracing an *overcoming disability* narrative) seemed absent from participants accounts of coping with ableism. The non-use of these strategies may be primarily due to there being no cure for cerebral palsy and attempting to deny or conceal the functional effects of the condition would be difficult for many. Participants tended to discuss collectivistic strategies (social creativity and social change). Examples included learning about social perspectives of disability (e.g., social model of disability), cognitively reframing ableist encounters as opportunities to reduce the chances of others experiencing ableism, identifying as a person with cerebral palsy, engaging in everyday activities, teaching people with cerebral palsy how to confront ableism, and participating in disability advocacy. Such examples highlight the importance for people with cerebral palsy of developing strong disability identities and of collective approaches to coping with ableism. Within participants’ descriptions of coping with ableism, there also seemed to be examples of high adaptive defences, including affiliation (turning to others for support), altruism (teaching others about confronting ableism), humour (emphasising the silliness of some forms of ableism), and suppression (avoiding thinking about ableist encounters). These defence mechanisms may serve to protect people with cerebral palsy from anxiety associated with common perceptions that they are *less than* others.

The relatively few examples of successfully confronting ableism captured in this research highlight the inherent challenges of such encounters. Participants provided few examples of successfully confronting ableism, and some adults with cerebral palsy declined to participate in this research because they were unable to recall any successful examples. Understood within the Confronting Prejudiced Responses Model (Ashburn-Nardo et al., 2008), it appears that many potential confrontations fail at any of the five hurdles. The first hurdle (interpreting an incident as discrimination) was sometimes not met when participants did not recognise interactions as ableism. The denial of their own cerebral palsy was one reason participants did not recognise ableism. An example of when the second hurdle (considering the discriminatory incident to be an emergency) may not have been met was when participants perceived that perpetrators probably had good intentions. Examples of circumstances contributing to the third hurdle (taking responsibility for responding) not being met include participants who questioned whether it was the responsibility of people with cerebral palsy to educate others or who were exhausted at having to explain themselves. The fourth hurdle (knowing how to respond) was sometimes not met due to participants feeling pressure to produce ideal responses. The fifth hurdle (deciding to take action) may not have been met when participants considered that attempts to confront ableism are generally unsuccessful.

The research findings could inform strategies to support people with cerebral palsy in their efforts to challenge the oppression they face. Evidence from the study—and the experiences of many of our team—is that ableism is poorly understood. Our findings suggest that people with cerebral palsy may benefit from information that helps them to recognise ableist microaggressions and equips them with practical strategies for responding. Such information could include clear definitions and examples of different types of ableism, approaches confronting and coping with these experiences, and case studies illustrating successful outcomes. Careful attention must be paid to ensuring this information is accessible, both in terms of content and dissemination. Alternative formats (e.g., audiovisual, plain language text, and Easy Read) and diverse dissemination channels warrant consideration to broaden reach. Our research, for example, has supported the development of plain language resources intended for publication on a national online cerebral palsy information platform. Peer support also offers a valuable avenue for sharing this information. Many of our team (the peer co-researchers) had been members of a peer support group for adults with cerebral palsy and spoke of the importance of surrounding themselves with like-minded people. In addition, learning about social perspectives of disability and receiving support from others with experiences of oppression were ways in which participants coped with ableism. These ways of coping with ableism could be drawn together in peer support groups, which may be able to foster the development of strong disability identities and the use of collective-level strategies for combating ableism.

Based on our findings, we suggest directions for future research. Greater clarity is needed around which strategies work, in what situations, and for whom. Our research seems to suggest that certain actions may be linked to specific confrontation outcomes but studies with larger samples are required to explore these potential connections. The *for whom* aspect acknowledges the diversity within the cerebral palsy population and highlights the need to adapt strategies to accommodate individual differences. Further insight is also needed into how information on confronting and coping with ableism might best be disseminated. We have prepared resources for online publication and highlighted the value of peer support. Generating evidence to guide what should be disseminated—such as the development and testing of resources—and how to distribute it would be worthwhile. Finally, attention must be paid to society-level strategies for confronting ableism. Our work focused on individual-level experiences of successfully confronting and coping with ableism with the intention of helping others to do similar. Collective action is also needed to drive change.

### Strengths and limitations

The key strength of this research was the leadership from people with cerebral palsy. With non-disabled researchers from health and social care disciplines typically driving the global research agenda on cerebral palsy, this project was an opportunity for adults with cerebral palsy to exercise self-determination and undertake research of interest and importance to their lives. People with cerebral palsy engaged in this project from the generation of the research topic to the dissemination of the findings.

This study also has limitations. First, we were unable to recruit as many participants as we intended. Some adults with cerebral palsy told us that they could not think of instances when they had successfully confronted ableism. These responses further highlight the difficulties with confronting ableism. Some may not have wished to participate because ableism is something they would rather forget about. Others were unwilling to participate without remuneration. Although it is common for people with disability to be paid as recognition of their valued contributions to research (Rickard & Purtell, 2011), our project was unfunded and, therefore, we were not able to remunerate participants. Second, we are unable to draw conclusions about whether experiences of ableism differ across the heterogeneous population of people with cerebral palsy. We did not collect data on participants’ functional abilities, and our sample size would not have been sufficient to support this type of analysis. Given evidence that perceptions of people with disability who confront ableism may vary by impairment type (Wang et al., 2019), there may be merit investigating such differences among people with cerebral palsy. Third, we encountered known challenges of critical participatory action research (Kemmis et al., 2014). The academic researchers were paid for their time but, due to no funding being available for the project, peer co-researchers with cerebral palsy were asked to volunteer their time. We attempted to address this challenge through ensuring the project was beneficial for them and minimised the burden of operational tasks on participants. We maximised decision-making opportunities, including the focus of the research and how the work was conducted. We set meeting times and project timelines that were suitable for the peer co-researchers, and carefully planned meetings to make them as meaningful as possible. Fourth, greater involvement and skill development of the peer co-researchers in data collection was desired but could not happen. Opportunities for involvement were limited due to: (a) the small number of interviews conducted, (b) some participants possibly being known to the peer co-researchers, and (c) some participants having limited times available to be interviewed.

## Conclusions

Ableism occurs frequently in the lives of people with cerebral palsy and is hard to confront. Despite the inherent challenges, people with cerebral palsy have, at times, successfully confronted ableism, bringing about changes in perpetrator behaviour (at the time) and apparent changes in perpetrator perceptions, preventing the recurrence of ableism, gaining insights into ableism and how it can be managed, and feeling successful. People with cerebral palsy have also found ways to cope successfully with the compounding effects of repeated occurrences of ableist microaggressions using adaptive coping mechanisms, developing strong disability identities, and engaging with collective-level strategies. Creating effective ways of supporting people with cerebral palsy to harness this knowledge needs to be prioritised so that we (people with cerebral palsy and our allies) are better positioned to challenge the social oppression people with cerebral palsy face.

## Supplementary Material

Supplementary material Gaskin_et_al_Supp_Table_1.docx

Supplementary material Gaskin_et_al_Supp_Table_2.docx

## Data Availability

The participants of this study did not give written consent for their data to be shared publicly, so, due to the sensitive nature of the research, supporting data is not available.
