# Supplementary material for: How adults with cerebral palsy successfully confront and cope with ableism: a peer-led research project
Source: Int J Qual Stud Health Well-being. 2026 Jan 15;21(1):2616117. doi: 10.1080/17482631.2026.2616117 (PMC12818316; doi:10.1080/17482631.2026.2616117)
Supplement: Supplementary material — Gaskin_et_al_Supp_Table_1.docx [file ZQHW_A_2616117_SM0552.docx]

**Supplementary Table 1**

*Researcher Reflexivity Statements*

| Researcher | Reflexivity Statement |
| --- | --- |
| Cadeyrn J. Gaskin | Moving and speaking differently to others, due to cerebral palsy, occasionally provokes ableist reactions towards me. In the latter half of my life, I have benefited from an academic understanding of social perspectives of disability, including ableism. Even with this knowledge, the typically strong feelings of indignation I experience when exposed to ableist microaggressions, coupled with my dysarthric speech, generally make me ineffective in responding to perpetrators. My insider status compromises my objectivity but also has benefits – I was easily able to build rapport with interviewees and to understand participants’ experiences. |
| Andrew D. Brown | I am a Queer man who tries to use my experiences challenging homophobia to build solidarity with all people experiencing oppression. As a social worker, I see it as a professional and ethical obligation to recognise the ways I benefit from being temporarily able bodied and my many other forms of privilege and to do my part in challenging all forms of ableist oppression. My research experience is all in community based and participatory research, and I brought that lens to this project as I do with all my work. |
| Sue Harris | I am a person with mild cerebral palsy who grew up in the 60'and 70's fully integrated into society. Having a lifetime career in disability, I have been mindful of many instances my clients have experienced ableism and have found myself frequently advocating on their behalf and educating perpetrators. Ironically, I must have been blissfully unaware of instances of ableism directed at me until I embarked on this research project. Thinking back, I wonder if my *normal* upbringing taught me to deflect ableism attacks. I have finally uncovered the scars and have gained valuable insight and understanding around ableism, its triggers, and ways to handle it. |
| Alex Birnie | As a woman with cerebral palsy, being involved in this research has given me a greater understanding of ableism and strategies with which to cope with it. In the past I haven't coped with ableism well, however this is now changing through advocating for myself and others. |
| Carmen Vargas | As a migrant woman from a developing non-English speaking country, I understand the effect of power dynamics, stigma and racism that happen on diverse people. I wanted to understand the challenges that people with cerebral palsy face and be a better ally. As an early career researcher with health promotion practice background my views on this research have been towards ideation of ongoing social change that could support others to be better allies for people with cerebral palsy. |
| Finn O’Keefe | I was born with mild cerebral palsy (spastic diplegia), which primarily affects the way I walk. While this marks me out as different and I have experienced ableism – as well as homophobia – in many forms, growing up I was encouraged not to talk too much about my disability. The term *ableism* is not even one I was aware of until recently. I have found learning about the experiences of others through this research extremely validating. It has helped me reflect on the often-unconscious way I deal with ableism in my daily life, and has changed my thinking about what successful responses can be. |
| Angela Dew | I have worked in the disability sector in Australia for over 40 years and have seen many welcome and necessary changes in the sector including recognition of the importance of hearing directly from those with lived experience of disability. Unfortunately, despite these changes, ableist attitudes persist in the community. This research helped me, as a disability scholar and fellow human being, to reflect on the everyday, insidious, and damaging nature of ableism and its impacts on the lives of people with cerebral palsy. |
| Debbie Dorfan | Moving differently to others due to cerebral palsy has meant I have both internalised and faced ableism. I see my disability acceptance as a journey and the more accepting I have become of my disability, the more I have been able to recognise and identify ableism, which helps me not internalise it, which makes it easier to be able to let slights and micro aggressions go and not be damaging. I am also continuing the long process of unpacking all the internalised ableism (having my self-identity formed at a time when there was not a term to define the concept) to live my best life the way I choose. |
| Freya E. Munzel | As a queer disabled woman my experiences of ableism and other forms of discrimination are unique to me. I think this helps me understand how diverse experiences with ableism can be in people with cerebral palsy. Being aware of my own experiences, and the biases these have formed, was necessary throughout this research. It is my hope that education such as this will assist adults with cerebral palsy and their allies. |
| Claudia Strugnell | As an educator and public health researcher who is a member of the LGBTIQ+ community, this research has provided me with the language to articulate and reflect on the damaging impact of ableism and marginalization. This awareness allows me to be a more informed and supportive ally for people living with cerebral palsy. |
| Maddie Fogarty | As a young disabled woman with cerebral palsy, I bring an insider perspective to the research, having personally experienced significant ableism throughout my life. These experiences have shaped my understanding of systemic barriers and prejudice faced by disabled people. My background allows me to connect deeply with participants' stories of discrimination, while also being mindful of how my own biases and coping mechanisms may influence how I interpret their experiences. |
| Adam Goodridge | As a man with cerebral palsy who is in a wheelchair, I have experienced ableism on many occasions, even if it is done through good intentions. The negative effects are not always recognized by others and, being nonverbal, it is often challenging to improve ableism where I see it. Being part of this research has helped me to identify ablism and discuss the issue with other researchers. |
| Joy Martin Mitchell | As an older generation person (born in 1970) who has direct lived experience of being a person who has cerebral palsy, the concept of ableism was a daily negotiation. Yet, I was specifically unaware of the term *ableism* and what it truly referenced, until being involved with this paper. Living with a disability and, at times, working on behalf of others who also have a disability, guaranteed a heightened awareness of any direct environment. Sometimes, in an effort to be realistic about a given situation, my own explanations involved an aspect of ableism, that may now be seen as abhorrent. Thus, as a person with disability, I would be guilty of voicing the direct reality of ableism. It did not automatically follow that I held the view I expressed, but rather, unconsciously, bringing the reality of ableism to the front of any discussion. Lived experience, and formal and informal peer advocacy, naturally reflected conscious and unconscious challenges and choices. Every day involved decisions about how to safely and meaningfully negotiate actively participating in the community. Ableism, known or otherwise, has always been part of my story. Like any contentious topic or issue within the community, if someone approaches a topic from a place of respect and furthering knowledge then, we can remain hopeful of constructive change within the general community. |
| Shelley Spencer | As a person with cerebral palsy, I have dealt with ableism all my life. This research paper has given me strategies to cope with ableism; it has also informed that people need to stand up against ableism. |
