# Supplementary material for: How adults with cerebral palsy successfully confront and cope with ableism: a peer-led research project
Source: Int J Qual Stud Health Well-being. 2026 Jan 15;21(1):2616117. doi: 10.1080/17482631.2026.2616117 (PMC12818316; doi:10.1080/17482631.2026.2616117)
Supplement: Supplementary material — Gaskin_et_al_Supp_Table_2.docx [file ZQHW_A_2616117_SM0556.docx]

**Supplementary Table 2**

*Summary of Successful Confrontations of Ableism*

| Ableism Type | Situation | Action | Successful Outcome |
| --- | --- | --- | --- |
| Perceived helplessness | A shopper tried to help the person with cerebral palsy enter a payment card personal identification number. | The person with cerebral palsy informed the shopper they were capable entering the personal identification number and requested space. | The shopper left the person with cerebral palsy to enter the personal identification number. |
| Perceived helplessness | A teacher wanted the person with cerebral palsy to use a substitute runner during a game. | The person with cerebral palsy told the teacher she did not need help and performed the task independently (ignoring the runner). | The teacher was surprised that the person with cerebral palsy could perform the task. |
| Perceived helplessness | The person with cerebral palsy’s father had low expectations for her. | The person with cerebral palsy ignored her father’s ableism, tried to prove him wrong, and substantively ended her relationship with him. | The person with cerebral palsy did not have to see her father. Her father admitted he was wrong about the person's ability to graduate university. The person achieved academically and realised she could not change her father’s ableism. |
| Spread effect | Nurses were apprehensive about how the person with cerebral palsy would be able to care for her newborn. | With her second and third newborns, the person with cerebral palsy was assertive and showed how she could care for her newborns. | The nurses realised that the person with cerebral palsy could care for her newborns. |
| Spread effect | A potential employer was concerned how the person with cerebral palsy would travel to and from work. | The person with cerebral palsy encouraged a former lecturer not to send students to the employer, given their ableism. | The lecturer was informed about the employer’s actions. |
| Spread effect | People whispered around the person with cerebral palsy, assuming the person could not hear. | The person with cerebral palsy asked them why they were whispering. | The people stopped whispering around the person with cerebral palsy. |
| Spread effect | People shouted at the person with cerebral palsy, assuming he could not hear. | The person with cerebral palsy responded that he was not deaf. | The people stopped shouting at the person with cerebral palsy. |
| Denial of privacy | A stranger enquired about the person with cerebral palsy’s *limp*. | The person with cerebral palsy explained to the stranger about cerebral palsy. | The stranger understood and asked no further questions. |
| Denial of privacy | A stranger enquired about the person with cerebral palsy’s *injury*. | The person with cerebral palsy bluntly responded that it was due to “a really bad birth injury” and walked away. | The person with cerebral palsy attributed this response to self-acceptance. |
| Denial of privacy | Following the delivery of training, a participant asked the person with cerebral palsy (the facilitator) how they toileted. | The person with cerebral palsy asked the same question of the participant, and each repeated the same question to the other multiple times. | The person with cerebral palsy did not answer the question, got to ask the same intrusive question, and the training participant appeared uneasy and walked away. |
| Denial of privacy | A bar patron asked the person with cerebral palsy, “What’s wrong with you, love?” | The person with cerebral palsy delivered a humorous, sexualised, verbal response. | The bar patron was surprised and left the person with cerebral palsy alone. |
| Denial of privacy | A stranger asked the person with cerebral palsy, “What’s wrong with you?” | The person with cerebral palsy politely refused to answer the question. | The person with cerebral palsy did not answer the question. |
| Denial of personal identity | School students referred to the person with cerebral palsy as “a spastic”. | The person with cerebral palsy told her parents what had occurred. | The students were made to apologise, and the class was educated about cerebral palsy. |
| Denial of personal identity | Mothers at the school gate assumed a support worker was the mother of the person with cerebral palsy’s child. | The person with cerebral palsy corrected the mothers that she was the child’s mother, and the other person was a support worker. | The mothers now knew that the person with cerebral palsy was the child’s mother and seemed confronted by this information. |
| Denial of disability | A school principal removed a bike’s training wheels against the person with cerebral palsy’s wishes. | Following an unsuccessful request for the principal to stop, the person with cerebral palsy told her parents. | The person with cerebral palsy’s parents confronted the principal. |
| Denial of disability | A stranger challenged the person with cerebral palsy about using an accessible parking space and prevented her from leaving. | The person with cerebral palsy told the stranger that she had a parking permit and showed the stranger how cerebral palsy affected her. | The stranger moved and the person with cerebral palsy was able to exit the car park. |
| Denial of disability | Adult children did not assist the person with cerebral palsy (their mother) with meal preparation. | The person with cerebral palsy started to refuse to do certain tasks. | The children assisted the person with cerebral palsy for a while. |
| Second-class citizenship | Waitstaff asked companions without disability questions instead of the person with cerebral palsy. | The person with cerebral palsy proactively redirected the attention of waitstaff towards herself and used clear and specific language. | The person with cerebral palsy was treated as an active participant. |
| Second-class citizenship | Conference organisers had not planned for the stage to be accessible. | The person with cerebral palsy emailed the organisers to request that the stage be accessible. | The conference organisers committed to improve accessibility in future. |
| Patronisation | Strangers referred to the person with cerebral palsy as “an inspiration” for achieving age-typical milestones. | The person with cerebral palsy explained that her achievements were not exceptional but were the result of her parents having similar expectation for her as their other children, and necessary support and resources. | The strangers generally responded well, but outcomes varied between strangers. |
| Patronisation | A stranger complemented the person with cerebral palsy for “coping well”. | The person with cerebral palsy retorted with a personal question about the stranger’s prostate. | The person with cerebral palsy confronted the stranger with a question as inappropriate as the stranger’s comment. |
| Patronisation | A potential employer patted the person with cerebral palsy on her arm instead of shaking her hand after she initially offered her left hand. | The person with cerebral palsy explained about her cerebral palsy during the job interview. | The person with cerebral palsy felt good about being able to explain her disability, learnt about how to handle similar situations in future, and realised people are not inherently bad or mean, but need educating. |
| Secondary benefits | A stranger followed the person with cerebral palsy into his workplace and asked to pray for him. | The person with cerebral palsy declined the stranger’s offer. | The stranger walked away. |
